# Cardiovascular effect of dental anesthesia with articaine (40 mg with epinefrine 0,5 mg % and 40 mg with epinefrine 1 mg%) versus mepivacaine (30mg and 20 mg with epinefrine 1 mg%) in medically compromised cardiac patients: A cross-over, randomized, single blinded study

**DOI:** 10.4317/medoral.17892

**Published:** 2012-02-09

**Authors:** Daniel Torres-Lagares, María Á. Serrera-Figallo, Guillermo Machuca-Portillo, José R. Corcuera-Flores, Carmen Machuca-Portillo, Raquel Castillo-Oyagüe, José L. Gutiérrez-Pérez

**Affiliations:** 1 Professor of Oral Surgery. Faculty of Odontology of Seville; 2 Associated Professor of Dentistry for Special Patients. Faculty of Odontology of Seville; 3 Professor of Dentistry for Special Patients and Periodontics. Faculty of Odontology of Seville; 4 Associated Professor. Faculty of Dentistry of Complutense University (Madrid)

## Abstract

Objectives: The aim of the present study is to compare cardiovascular safety profiles of two dental anesthetics: articaine versus two standard mepivacaine solutions used during etiological periodontal treatment in cardiovascular patients. Study Design: Using a cross-over study design, ten cardiovascular patients were randomly assigned to dental treatment with 1.8mL of a local anesthetic injected on each quadrant of the mouth: Articaine (40mg with Epinephrine 0.5mg % and 40mg with Epinephrine 1mg %) or Mepivacaine (30mg and 20mg with Epinephrine 1mg %). A computer programme enabled continuous longitudinal data collection: O2 saturation, blood pressure (BP) and heart rate (HR). Results: No severe clinical side effects were observed. During the treatment period, we observed statistically significant differences as regards HR between injections with and without adrenalin (p< 0.039) and as regards systolic (p< 0.046) and diastolic (p < 0.046) blood pressure during the stabilization period. In both cases, the parameters under study increase. Age, gender, jaw treated, treatment duration and the rest of cardiovascular variables did not affect the results. None of the patients underwent ischemic alterations or any other complication derived from the treatment or the anesthesia. Conclusions: According to the results of our study, dental anesthetics with standard concentrations of Epinephrine seem to alter HR and BP. Although no cardiac ischemic alterations or any other cardiovascular complications have been observed, we must be cautious with the administration of anesthetics containing vasoconstrictors in patients with cardiovascular diseases.

** Key words:**Dental anesthesia, cardiovascular diseases, chronic periodontitis, drug toxicity.

## Introduction

An essential factor which determines a successful causal periodontal treatment is the administration of good-quality local anesthesia (LA). Though mepivacaine has been traditionally used as the dental anesthetic of choice, clinically potent local anesthetic substances, such as mepivacaine and articaine, are used in combination with vasoconstrictors. Vasoconstrictors, mainly adrenaline or epinephrine, contribute to successful LA as they increase the depth and duration of analgesia. Such effect of epinephrine on mepivacaine or articaine-based LA solutions has already been demonstrated. (1) Vasoconstrictors also promote hemostasis and are routinely incorporated in most commercial preparations. (2-4) Furthermore, by concentrating the LA agent at the infiltration site, the vasoconstrictor diminishes the risk for systemic side effects of LA.

Physiological responses associated with local anesthetics containing a vasoconstrictor include changes in heart rate and blood pressure (5-7), dysrrhythmias (8,9), ischemic alterations (10,11), endogenous catecholamines release (12), endocrine response to surgery, and hypokalemia (13,14). These changes are regulated by the net balance between sympathetic and parasympathetic activity, and both stress and pain will further modify autonomic response (15,16).

When these vasoconstrictor-induced physiological responses exceed the normal range, the risk of morbidity or even mortality increases. This is of special relevance in the case of cardiovascular patients. Although most current literature accepts that adrenaline has a safety range (17,18), its threshold in cardiovascular patients is not clear yet. (19,20)

Although the information available as regards the cardiovascular response to dental LA with articaine and epinephrine and with mepivacaine with epinephrine is limited to healthy patients (21,22), it may still be of value to cardiologists, primary care physicians, surgeons and dentists when it comes to select a local anesthetic solution for cardiovascular patients.

The purpose of the present study is to examine the hemodynamic response to four LA solutions in cardiovascular patients: Articaine 40mg with epinephrine 0.5mg %, articaine 40mg with epinephrine 1mg %, mepivacaine 30mg without epinephrine and mepivacaine 20mg with epinephrine 1mg %. The hypothesis is that the synergy of a short half-life time LA agent with a proper concentration of epinephrine would be safer for cardiovascular patients.

## Material and Methods

Study design and patients selection

A prospective, randomized, single-blinded, crossover, controlled comparative study was performed at the Department of Special Care in Dentistry, Seville University, Faculty of Odontology during 2009. The study was conducted with the approval of the University of Seville Ethics Committee and in accordance with the Declaration of Helsinki.

All patients gave their informed consent to take part in the study prior to the initiation of the dental treatment. Randomization was achieved by using a computer-generated random number list. Ten patients with cardiovascular disease who met the inclusion criteria were enrolled in the study.

The inclusion criteria were the following: (a) controlled hypertension (HTN) with blood pressure values ≤ 160/100 mm Hg; (b) ischemic heart disease (IHD) including stable angina pectoris, post-myocardial infarction (≥ 6 months); (c) chronic periodontitis affecting the four quadrants of the oral cavity.

Exclusion criteria comprised: (a) cardiovascular instability including unstable angina pectoris, recent myocardial infarction (≤ 6 months), refractory dysrrhythmias, untreated or uncontrolled hypertension, untreated or uncontrolled CHF, uncontrolled hyperthyroidism; (b) uncontrolled diabetes mellitus; (c) sulphite sensitivity; (d) steroid-dependent asthma; (e) pheochromacytoma; (f) tricyclic antidepressant treatment; (g) previous history of psychiatric disorder, chronic use of central nervous system depressants or antidepressants or mental instability.

All patients underwent clinical and radiographic examination (periodontal charting and orthopantomography). Patients who presented with chronic periodontitis affecting the four quadrants of the oral cavity treatable with etiological periodontal therapy (root planning) were randomly allocated in order to determine the order in which each of the anesthetics would be applied to the different quadrants. Root planning was programmed following a weekly schedule for each of the quadrants. In each of the treatment arms, a different LA solution was used: (a) mepivacaine 30 mg without epinephrine (Normon, Spain) total dose: 108 mg of mepivacaine chlorhydrate; (b) articaine 40 mg with epinephrine 0.5mg% (Normon, Spain) total dose: 144 mg articaine hydrochloride with 0.018 mg of epinephrine; (c) articaine 40 mg with epinephrine 1 mg% (Normon, Spain) total dose: 144mg articaine hydrochloride with 0.036mg of epinephrine, and (d) mepivacaine 20mg with epinephrine 1mg% (Inibsa, Spain) total dose: 72mg mepivacaine chlorhydrate with 0.036 mg of epinephrine. We used a cross-over study design with a washing period of one week.

Treatment protocol

The study time frame for each subject comprised a baseline period of 5 minutes followed by LA injection. Anesthesia was carefully induced with aspiration and slow injection of anesthetic. All patients were administered a standard dose of 3.6 mL. A minimum of 5 minutes was necessary to achieve LA effectiveness. Treatment lasted until completion of dental procedure (one session of root planning and polishing per quadrant). Patients were asked to remain seated for several minutes after the completion of the treatment. The same two investigators attended every treatment session, one of them in charge of the treatment and the other one in charge of monitoring and data recording.

Monitoring 

A non-invasive monitor (Avant 2020 pulsioximeter, Nonin Medical Inc. Minnessotta, USA) was used to record systolic, diastolic and mean blood pressure (sysBP, diaBP, meanBP, respectively) at 5-minute intervals. Oxygen blood saturation (SO2) and heart rate (HR) were continuously recorded. Online transmission of data from the pulsioximeter to a personal computer was mediated with software nVISION v. 5.1e (Nonin Medical Inc. Minnessotta, USA). Monitoring began at the onset of the baseline period and continued every 5 minutes after completion of the periodontal treatment.

Statistics

Standard time points and time intervals were defined: (a) Baseline: beginning of monitoring; (b) Baseline + 5 minutes: end of stabilization period; (c) LA: injection timing; (d) LA + 5 minutes: 5 minutes after injection; (e) Treatment: onset of dental treatment; (f) End: completion of treatment.

The hemodynamic indices measured at the above mentioned points were used to carry out the descriptive statistic analysis (means, SD). Demographic parameters and hemodynamic indices of the 4 treatment arms were compared using t-test and analysis of variance (ANOVA, Bonferroni post-hoc test). Kolmogorov-Smirnov test was used to confirm the normal distribution of data.

## Results

Baseline characteristics of patients were compared in the two groups as regards mean age (63±12.5 yrs), jaw under treatment and duration of treatment (26.38±9.9 minutes).

Programmed dental procedure was completed in all 10 patients. No severe adverse effects were observed in any of the patients. Clinical data of patients are shown in ( Table 1).

**Table 1:**
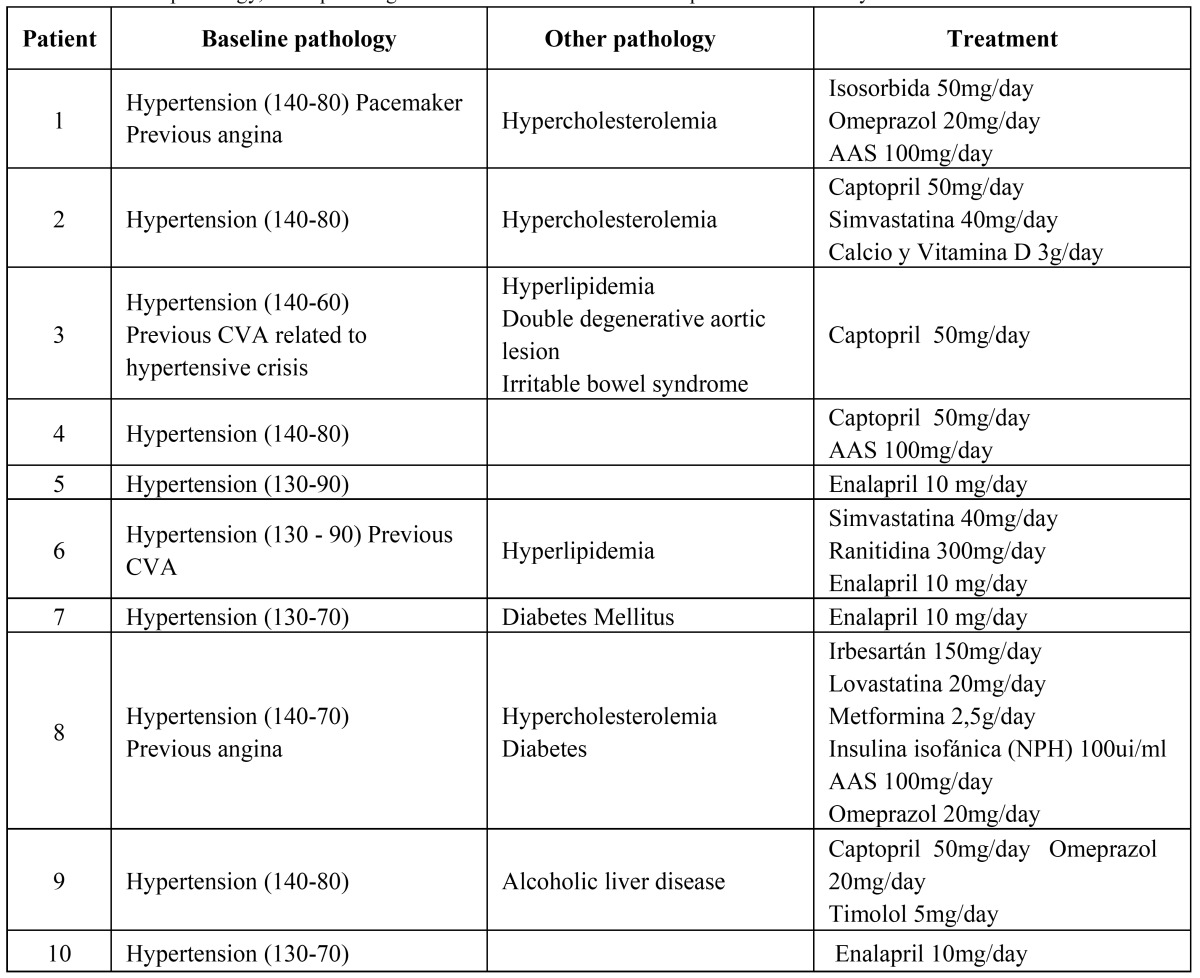
Baseline pathology, other pathologies and baseline treatment in the patients under study.

Hemodynamic indices of HR, sysBP and SO2 at various time points and time intervals showed no statistical differences when comparing both groups. The group receiving Mepivacaine 2% with epinephrine 1:100,000 showed statistical differences with the rest of the groups as we observed increased diaBP levels at the stabilization period (p< 0.038) and meaBP (p< 0.01) at the stabilization period and in the treatment period (p< 0.039) ( Table 2).

**Table 2:**
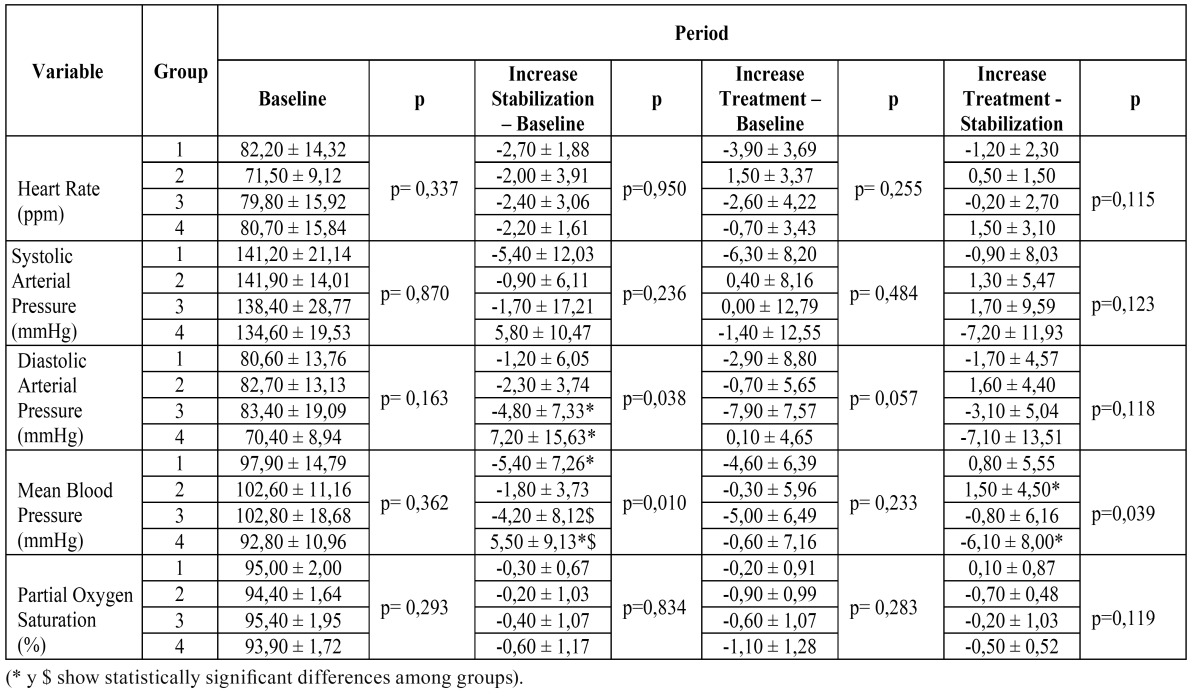
Values of the different variables studied in the four study groups during baseline, stabilization and treatment period. (Groups: 1.- Mepivacaine chlorhydrate at 3% 2.- Articaine hydrochloride 40 mg + epinephrine 5mg% 3.- Articaine hydrochloride 40 mg + epinephrine 1 mg% 4.- Mepivacaine chlorhydrate at 2% + epinephrine 1:100.000; n=10 in all of them).

To analyse the effect of epinephrine on the anesthetics, clinical data were divided into two study groups: with (n= 30) and without (n=10) adrenaline. Hemodynamic indices of HR (p< 0.039), sysBP (p< 0.046), diaBP (p< 0.046), at various time points and time intervals (treatment and stabilization periods) were statistically different in both groups ( Table 3).

**Table 3:**
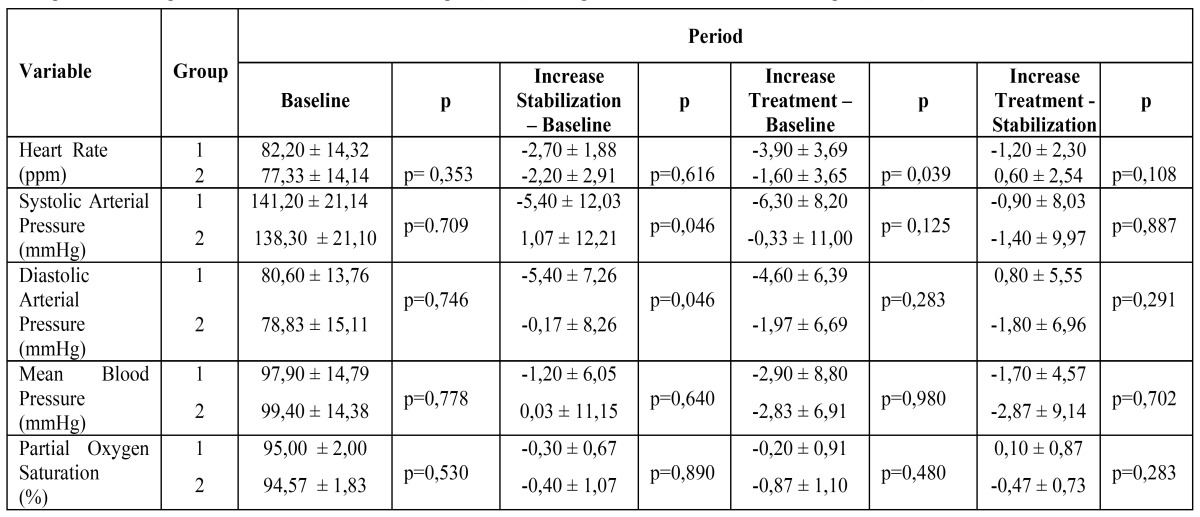
Values of the different variables in the groups of anesthetics with and without vasoconstrictors during baseline, stabilization and treatment periods. Group without vasoconstrictor = Group 1 (n=10); Group with vasoconstrictor = Group 2 (n = 30).

## Discussion

The use of local anesthetics associated to vasoconstrictors is somewhat controversial among the professionals of odontology because, together with the beneficial effects of such combinations, other side effects have been detected, mainly of cardiovascular nature (5-7).

Such side effects may be adverse, although the use of these substances is not contraindicated in healthy subjects whenever the adequate doses and correct administration procedure is followed. In fact, most studies on the alterations of vascular parameters associated with the use of dental anesthetics containing vasoconstrictors have been carried out in healthy subjects, which would explain the possible bias observed in the results (23-27).

In Western civilization, the number of cardiovascular patients who visit a dental clinic is increasing. Very few studies have so far analysed the effect of dental anesthetics containing vasoconstrictors on cardiovascular patients (28,29). Therefore, it is of the utmost importance to determine, by means of controlled trials, the effect such substances may have on this type of patients in order to allow the safe use of anesthetics with vasoconstrictors in a greater number of patients.

The criterion used to group patients with cardiovascular diseases is not homogeneous in the different studies published so far. The present study analyses a 100% of patients with arterial hypertension. Conrado et al. (28) study a sample of 100% of patients with heart failure. Elad et al. (29) do not report the disease rate in their sample of patients, but use controlled arterial hypertension as inclusion criteria.

In the present study, alterations in heart rate were not significant when we compared the four study groups. Nevertheless, when we compared the use of anesthetics without and with vasoconstrictors, in the first group we observed a significant decrease in heart rate in comparison to the second group. This effect is similar to the one reported by Matthews et al. (30), who detected a decrease of 10 ppm. In our sample, we have observed a reduction of 4 ppm. In any case, heart rate does not increase above baseline levels and therefore it always remains within a safe interval throughout the treatment period.

As regards arterial pressure, we have observed few significant alterations, mainly referring to an increase of diastolic arterial pressure and mean blood pressure during the stabilization period in comparison with baseline levels. Diastolic arterial pressure and mean blood pressure increased significantly in group 4 (mepivacaine chlorhydrate at 2% + epinephrine 1:100,000) in comparison to the rest of groups. If we focus on the first three study groups (mepivacaine chlorhydrate at 3%; 40 mg articaine hydrochloride + 0.5 mg% epinephrine; 40mg articaine hydrochloride + 1mg % epinephrine), the alterations detected are similar to those reported by Conrado et al. (28), Neves et al. (31) or Elad et al. (29). Increases observed in the fourth group (mepivacaine chlorhydrate at 2% + epinephrine 1:100,000) have not been reported in the literature as this type of anesthetic has not been analysed. As a result, we must be cautious when administering this anesthetic combination to cardiovascular patients.

When we classify the results obtained according to the use or not of vasoconstrictors, we observed alterations in diastolic and systolic blood pressure between the baseline period and the stabilization period. This would confirm the critical nature of this period, already observed in previous reports (28,31).

As regards oxygen partial pressure, our results do not show remarkable alterations when comparing the different study groups, similarly to what Matthews et al. (30) had already reported.

Finally, we must add some points about the different treatment regimes analysed. All of them have been well tolerated by patients. Even in the case of cardiovascular patients, some of them suffering from more than one disease, no adverse side effects have been detected. As a result, and considering the results obtained, we can confirm that these treatments are very safe in all cases.

Yet, as has been clearly demonstrated, the combination of anesthetics and vasoconstrictors provokes cardiovascular alterations which could have serious clinical consequences in patients with more severe pathologies than the ones here studied.

It would be advisable to increase the level of complexity of the techniques used to obtain data (use of Holter, electrocardiogram, cardiac enzyme study, etc.) in order to identify ischemic events of short duration. However, we should also justify the necessity of these complex techniques.

In our opinion, more complex studies would be justified in the case of severe cardiovascular patients as alterations which are unimportant in the case of healthy subjects could have clinical relevance in these patients. Nevertheless, such studies should be accompanied by greater safety measures in the case of patients more severely compromised.
